# C-Kit receptor and tryptase expressing mast cells correlate with angiogenesis in breast cancer patients

**DOI:** 10.18632/oncotarget.23722

**Published:** 2017-12-22

**Authors:** Ilaria Marech, Michele Ammendola, Christian Leporini, Rosa Patruno, Maria Luposella, Nicola Zizzo, Giuseppe Passantino, Rosario Sacco, Ammad Ahmad Farooqi, Valeria Zuccalà, Silvana Leo, Rosalba Dentamaro, Mariangela Porcelli, Pietro Gadaleta, Giovambattista De Sarro, Cosmo Damiano Gadaleta, Girolamo Ranieri

**Affiliations:** ^1^ Interventional and Medical Oncology Unit, National Cancer Research Centre, Istituto Tumori Giovanni Paolo II, 70124 Bari, Italy; ^2^ Department of Medical and Surgery Science Medical School, Clinical Surgery Unit, Magna Graecia University, 88100 Catanzaro, Italy; ^3^ Department of Health Science, Clinical Pharmacology and Pharmacovigilance Unit, Pharmacovigilance's Centre Calabria Region, Magna Graecia University, Germaneto, 88100 Catanzaro, Italy; ^4^ Chair of Pathology, Veterinary Medical School, Aldo Moro University, 70010 Valenzano, Italy; ^5^ Cardiovascular Disease Unit, San Giovanni di Dio Hospital, 88900 Crotone, Italy; ^6^ Laboratory for Translational and Personalized Medicine, Rashid Latif Medical College, University of Lahore, 44000 Islamabad, Pakistan; ^7^ Pathology Unit, Pugliese-Ciaccio Hospital, 88100 Catanzaro, Italy; ^8^ Medical Oncology Unit, Vito Fazzi Hospital, Piazzetta Muratore, 73100 Lecce, Italy; ^9^ Senology Unit, National Cancer Research Centre, Istituto Tumori Giovanni Paolo II, 70124 Bari, Italy

**Keywords:** C-Kit receptor, tryptase, mast cells, angiogenesis, breast cancer

## Abstract

C-Kit protein is a transmembrane tyrosine kinase (TK) receptor (c-KitR-TK), which is predominantly expressed on mast cells (MCs) playing a role in tumor angiogenesis. It could be also expressed on epithelial breast cancer cells (EBCCs), but no data have been published regarding the correlation between mast cells positive to c-KitR (MCs-c-KitR), EBCCs positive to c-KitR (EBCCs-c-KitR), BC angiogenesis in terms of microvessel density (MVD) and the main clinic-pathological features. This study aims to evaluate the above parameters and their correlations in a series of selected 121 female early BC patients. It has been found a strong correlation between MVD and MCDPT, and MCs-c-KitR, MVD and MCs density positive to tryptase (MCDPT), and MCs-c-KitR and MCDPT by Pearson correlation. These data suggest an involvement of both MCDPT and MCs-c-KitR in BC tumor angiogenesis. Furthermore, BC tissue expressing c-KitR could be a putative predictive factor to c-KitR-TK inhibitors. In this way, selected patients with higher MCs-c-KitR could be candidate to receive c-KitR-TK inhibitors (e.g. masitinib, sunitinib) or tryptase inhibitors (e.g. nafamostat mesilate, gabexate mesilate).

## INTRODUCTION

C-KitR is the transmembrane tyrosine kinase (TK) receptor (c-KitR-TK) for stem cell factor (SCF), a cytokine regulating important functions of MCs such as proliferation and degranulation that in turn stimulate angiogenesis [1, 2]. C-KitR could be expressed also in other stromal cells, such as myofibroblasts and adipocyte cells, and epithelial breast cancer cells (EBCCs) stimulating proliferation. Several published studies showed that EBCCs positive to c-KitR (EBCCs-c-KitR) are low (from 10% to 29%) or absent in invasive breast cancer (IBC) [3–11]. In agreement, a progressive decrease and almost complete loss of EBCCs-c-KitR have been reported during the progression of normal tissue to BC [7, 8, 11–13]. Furthermore, EBCCs-c-KitR have been reported to be associated with more indolent cancer behavior [3, 6]. However, a small body of evidence suggests that EBCCs-c-KitR might represent an independent negative prognostic factor [4, 5, 10, 14–17]. Notably, c-KitR overexpression in IBC appeared to be an indicator of high-grade cancer resulting in poor prognosis [18]. This evidence suggests that c-KitR expression may play a role in BC progression. C-KitR expression has been also found increased in malignant breast phyllodes tumors (BPT), uncommon stromalepithelial lesions with different potential of malignancy [7, 8, 19–25]. For what concern the role of EBCCs-c-KitR and tumor angiogenesis, very little data have been published and only one study explored the relationship between EBCCs-c-KitR and microvascular density (MVD) demonstrating a negative correlation [3]. With special regard to MCs positive to c-KitR (MCs-c-KitR), a lot of *in vitro* data demonstrated that MCs play a role in tumor angiogenesis [26–31]. In particular, MCs stimulate angiogenesis by several mechanisms including c-KitR activation leading to the release of a plethora of angiogenic factors, contained in their cytoplasmic secretory granules [32, 33]. Among them, the most powerful factor is tryptase [32, 34]. Tryptase, acting on the proteinase-activated receptor-2 (PAR-2) by its proteolytic activity, has angiogenic activity stimulating both human vascular endothelial and tumor cell proliferation in paracrine manner, helping tumor cell invasion and metastasis [30, 35]. In *in vivo* studies it has been also shown that MCs density positive to tryptase (MCDPT) is strongly related to angiogenesis in several animal and human malignancies [2, 28, 33, 36–50]. With concern to early BC patients, we already demonstrated a strong correlation between high serum tryptase levels before surgery (STLBS) and MVD, STLBS and MCDPT, MCDPT and MVD [40].

To the best of our knowledge, no data have been published regarding the correlation between EBCCs-c-KitR, MCs-c-KitR, BC angiogenesis and clinico-pathological features. In the current study we aim to evaluate the primary tumor tissue status of the above parameters to perform any possible correlation to each other and with clinico-pathological characteristics in a series of 121 female early BC patients. Adjacent normal breast tissue has been also evaluated in terms of MCDPT, MVD, MCs-c-KitR and normal breast epithelium-c-KitR (NBE-c-KitR) expression. Finally, difference between the all the evaluated parameters in tumor tissue and adjacent normal breast tissue has been also assessed.

## RESULTS

**Figure 1:**
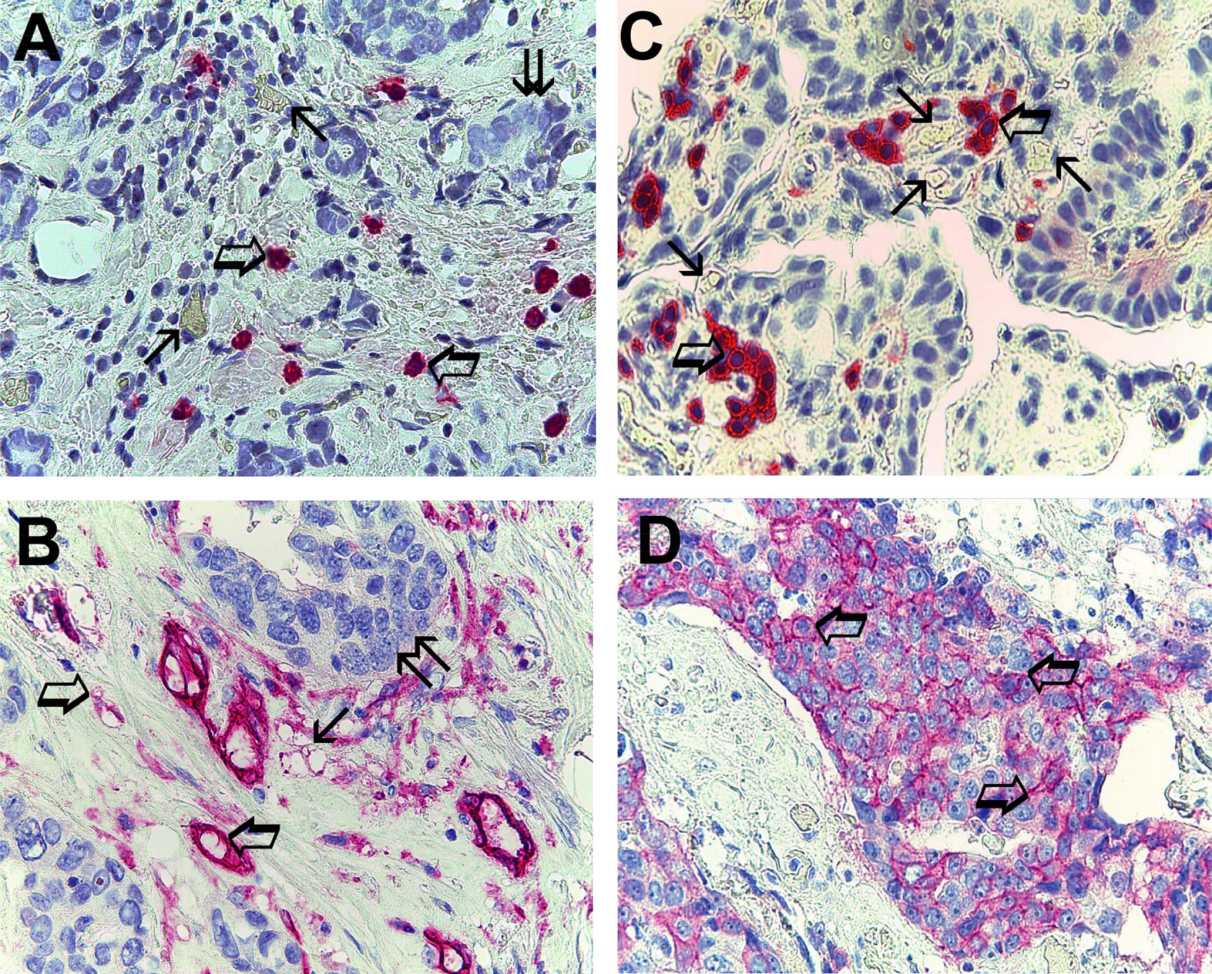
(**A**) Breast cancer tissue sections evaluated by immunohistochemistry with the primary anti-tryptase antibody. Big arrows indicate single scattered red immunostained tryptase-positive mast cells, small arrows indicate single microvessels with red blood cells in theirs lumen and finally twice arrow indicates a cluster of breast cancer cells. Original magnification: x 400. (**B**) Breast cancer tissue sections evaluated by immunohistochemistry with the primary anti-CD34 antibody. Big arrows indicate single scattered red immunostained microvessel, small arrow indicates a cluster of red immunostained microvessels and finally twice arrow a cluster of breast cancer cells. Original magnification: x 400. (**C**) Breast cancer tissue sections evaluated by immunohistochemistry with the primary anti-cKitR antibody. Big arrows indicate single scattered red immunostained c-KitR positive mast cells with a well evident membranous staining. Small arrows indicate single microvessels with red blood cells in theirs lumen. Original magnification: x 400. (**D**) Breast cancer tissue sections evaluated by immunohistochemistry with the primary anti-cKitR antibody. Many red immunostained epithelial breast cancer cells positive to c-KitR. Big arrows indicate a well evident positive c-KitR membranous staining. Original magnification: x 400.

Data obtained from tumor tissue using light microscopy and image analysis system (Quantimet500 Leica, Wetzlar, Germany) [33] show the following mean ± 1 s.d.: MCDPT 7.49±2.81 (Figure 1A), MVD 29.41 ± 6.63 (Figure 1B), MCs-c-KitR 8.75 ± 3.26 (Figure 1C) and EBCCs-c-KitR 32.98 ± 16.61 (Figure 1D) (Table 2). MCs appear as round or spheroidal cells with a diffuse cytoplasmic red staining using the anti-tryptase antibody and with a filiform peripheral cell membranous intense staining using the anti-c-KitR and a blue spheroidal central nucleus. MCs are found as scattered cells (Figure 1A) or as cluster formation near and around microvessels that sometimes showed several red blood cells in their lumens (Figure 1C). Furthermore, microvessels appear as red immunostained structures and often their lumen is visible using the anti-CD34 antibody (Figure 1B). With special reference to EBCCs, a part of them shows a red strong filiform membranous staining utilizing the anti-c-KitR antibody (Figure 1D).

**Figure 2:**
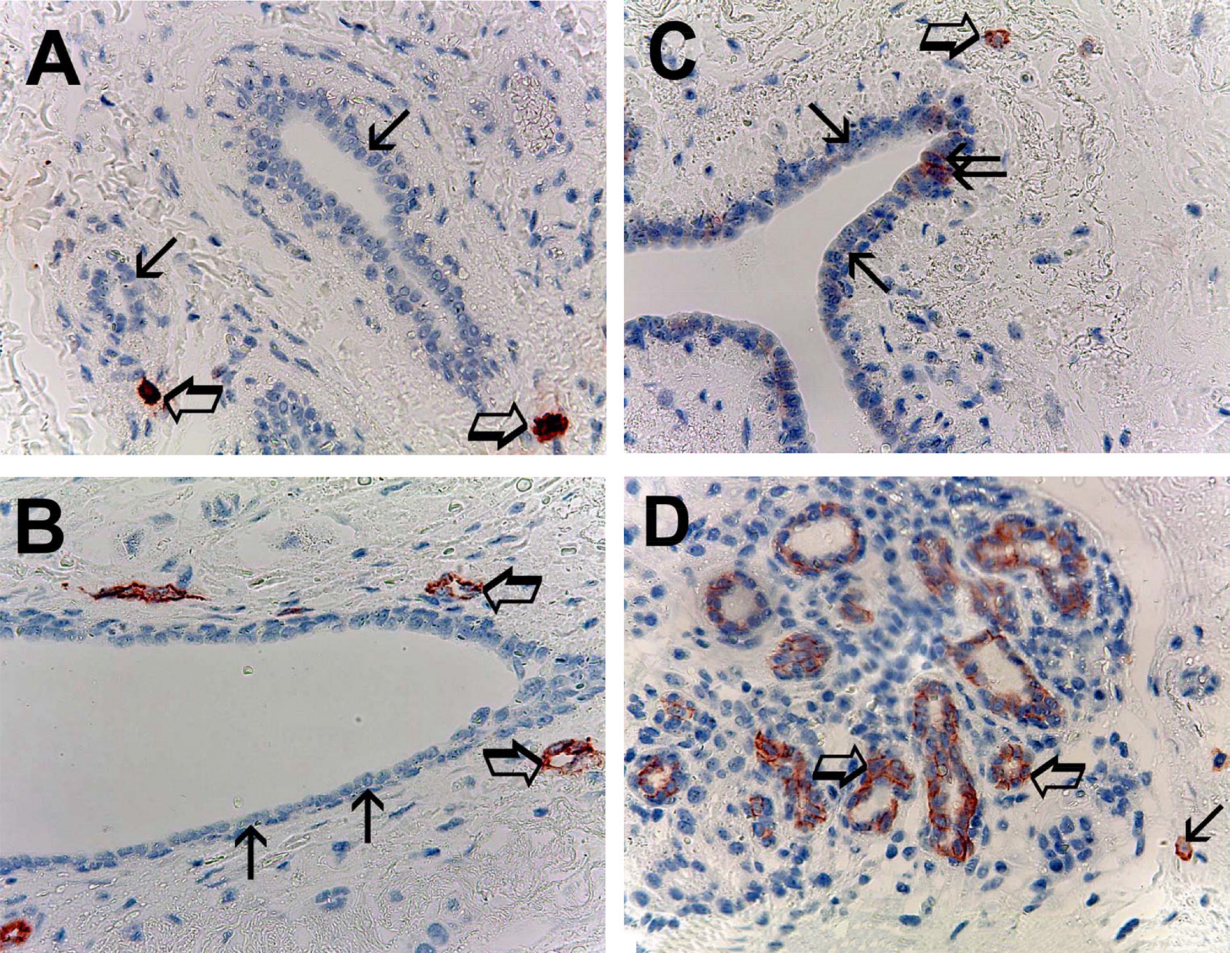
(**A**) Breast normal tissue sections evaluated by immunohistochemistry with the primary anti-tryptase antibody. Big arrows indicate only two single scattered red immunostained tryptase-positive mast cells in all examinated field. Small arrows indicate normal breast epithelial cells. Original magnification: x 400. (**B**) Breast normal tissue sections evaluated by immunohistochemistry with the primary anti-CD34 antibody. Big arrows indicate two single scattered red immunostained microvessels, small arrows indicate normal breast epithelial cells. Original magnification: x 400. (**C**) Breast normal tissue sections evaluated by immunohistochemistry with the primary anti-cKitR antibody. Big arrow indicates a single mast cell with the red filiform membranous staining and its central blue nucleus. Small arrows indicate normal breast epithelial cells negative to cKitR immunostaining. Twice arrow indicates two normal breast epithelial cells positive to cKitR immunostaining. Original magnification: x 400. (**D**) Breast normal tissue sections evaluated by immunohistochemistry with the primary anti-cKitR antibody. Big arrows indicate normal breast epithelial cells positive to cKitR immunostaining. Small arrow indicates a mast cell red immunostained. Original magnification: x 400.

With special regard to normal tissue [33] the evaluated parameters showing the following mean ± 1 s.d.: MCDPT 2.86 ± 1.24 (Figure 2A), MVD 12.38 ± 3.97 (Figure 2B), MCs-c-KitR 3.01 ± 1.57 (Figure 2C) and EBCCs-c-KitR 59.69 ± 27.35 (Figure 2D) (Table 2).

In normal tissue EBCCs show the red strong filiform membranous staining utilizing the anti-c-KitR antibody; interestingly the part of EBCCs negative to the same immunostaining represents the internal negative control (Figure 2C, 2D). Obtained data evaluated by *t*-test analysis show a significant difference between mean regarding tumor tissue vs normal tissue in terms of MCDPT (*p* = 0.001), MVD (*p* = 0.003) MCs-c-KitR (*p* = 0.001), EBCCs-c-KitR vs NBE-c-KitR (*p* = 0.02) as summarized in Table 2.

**Figure 3:**
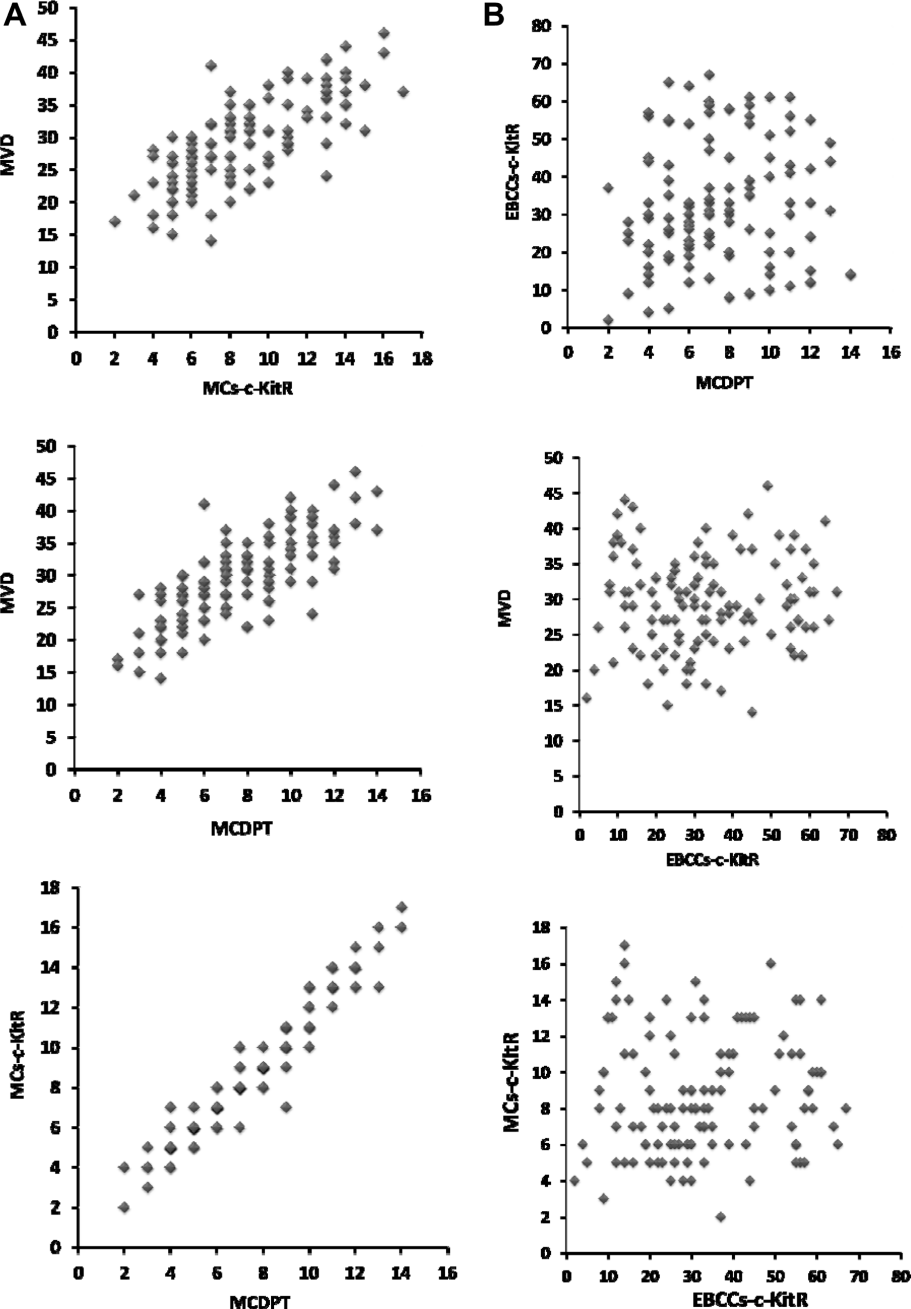
(**A**) Correlation analysis between: MVD and MCs-c-KitR (r = 0.77, *p* = 0.001), MVD and MCDPT (r = 0.83, *p* = 0.000), and MCs-c-KitR and MCDPT (r = 0.94; *p* = 0.000). (**B**) Correlation analysis between: EBCCs-c-KitR and MCDPT (r = 0.067, *p* = n.s.), MVD and EBCCs-c-KitR (r = 0.18, *p* = n.s.), c MCs-c-KitR and EBCCs-c-KitR (r = 0.042; *p* = n.s.).

A significant correlation between MVD and MCs-c-KitR (r = 0.77, *p* = 0.001), MVD and MCDPT (r = 0.83, *p* = 0.000), and MCs-c-KitR and MCDPT (r = 0.94; *p* = 0.000) is found by Pearson correlation (Figure 3A). There is no correlation between EBCCs-c-KitR and MCDPT (r = 0.067, *p* = n.s.), MVD and EBCCs-c-KitR (r = 0.18, *p* = n.s.), MCs-c-KitR and EBCCs-c-KitR (r = 0.042; *p* = n.s.) by Pearson test (Figure 3B).

## DISCUSSION

C-KitR, also known as CD-117 (according to cluster differentiation nomenclature) is the receptor for SCF, a cytokine regulating important functions of MCs such as proliferation and degranulation that in turn stimulate angiogenesis [1, 2]. In particular, c-KitR-mediated MCs activation has been shown to induce tumor angiogenesis by the release of tryptase, the most powerful pro-angiogenic factor stored in MCs granules. In a pre-clinical model tryptase induces *in vitro* endothelial cells (ECs) proliferation in a matrigel assay and displayed *in vivo* capillary growth in the chick embryo chorioallantoic membrane, which was suppressed by tryptase inhibitors. In addition, tryptase can act on PAR-2 that can be expressed on epithelial cells stimulating cell proliferation of cancer cells in paracrine manner, potentiating invasion and metastasis [27–32, 51].

**Table 1:** MCDPT, MVD, MCs-c-KitR and EBCCs-c-KitR as a function of clinico-pathological characteristics in a series of 121 breast cancer patients

Variable	No. of patients	No. of tumours with high MCDPT^a^ (%)	No. of tumours with high MVD^b^(%)	No. of tumours with high MCs-c-KitR (%)^c^	No. of tumours with high EBCCs-c-KitR (%)^d^	No. of tumours with high STLBS ** (%)
*Age, years*						
Range 26–87	121					
Median 57	121					
< 57years	56	29 (52)	30 (54)	33 (59)	27 (48)	26 (55)
≥ 57 years	65	37 (57)	34 (52)	32 (49)	33 (51)	31 (54)
*Menopausal status*						
Premenopausal	51	28 (55)	24 (47)	27 (53)	29 (57)	23 (53)
Postmenopausal	70	38 (54)	37 (53)	39 (56)	34 (49)	32 (52)
*Hystological type*						
Ductal	88	40 (45)	47 (53)	43 (49)	44 (50)	38 (49)
Lobular	33	17 (52)	16 (48)	15 (45)	19 (58)	13 (48)
*Tumour size*						
pT_1_	67	36 (54)	32 (48)	35 (52)	33 (49)	27 (55)
pT_2_	36	17 (47)	20 (56)	18 (50)	19 (53)	18 (51)
pT_3_	18	8 (44)	9 (50)	10 (56)	9 (50)	10 (50)
*Nodal status*						
pN_0_	52	23 (44)	25 (48)	28 (54)	24 (46)	20 (46)
pN_1–2_	69	34 (49)	37 (54)	33 (48)	31 (45)	33 (54)
*Cytohistological grade*						
G_1_	42	23 (55)	19 (45)	21 (50)	22 (52)	21 (58)
G_2_	51	24 (47)	23 (45)	25 (49)	24 (47)	21 (47)
G_3_	28	16 (57)	13 (46)	15 (54)	12 (43)	11 (50)
*Estrogen receptor status*						
Negative	32	17 (53)	18 (56)	14 (44)	19 (59)	13 (46)
Positive	89	44 (49)	47 (53)	48 (54)	42 (47)	36 (48)
*Progesteron receptor status*						
Negative	38	19 (50)	16 (42)	18 (47)	21 (55)	18 (53)
Positive	83	36 (43)	37 (45)	39 (47)	45 (54)	39 (55)
*c-erbB-2 status*						
Negative	84	37 (44)	44 (52)	45 (54)	40 (48)	35 (51)
Positive	37	22 (59)	20 (54)	16 (43)	17 (6)	19 (52)

^a^Median cut-off value: 7 cells per 400 field.

^b^Median cut-off value: 29 microvessels per 400 field.

^c^Median cut-off value: 8 cells per 400 field.

^d^Median cut-off value: 31 cells per 400 field.

*In vivo* studies indicate that MCDPT is strongly related to angiogenesis in several malignancies [28, 33, 36–41]. In particular, pilot data suggest that MCDPT play a role in early BC angiogenesis [52]. Results from Ribatti et al. demonstrated that MCDPT contribute to angiogenesis leading to lymph nodes micrometastases in BC patients [53]. In agreement with the above literature, we have already demonstrated an involvement of increased circulating tryptase and MCDPT in BC angiogenesis [40].

With special regard to IBC, several evidences suggested that EBCCs-c-KitR are low (from 10% to 29%) or absent [3–11]. Consistently, a progressive decrease and almost complete loss in EBCCs-c-KitR have been showed during the progression of normal tissue to BC [11–13]. In fact, c-KitR is expressed by non-malignant epithelial rather than malignant epithelial breast tissue employing immunohistochemistry [7, 8]. Furthermore, EBCCs-c-KitR are related to a more indolent cancer behavior: high disease free survival, low grading, metastatic lymph nodes negative, receptor (ER, PgR, HER2neu) positive status [3, 6]. Conversely, some studies demonstrated that EBCCs-c-KitR may represent an independent factor of poor prognosis [5], as they are associated with receptor (ER, PgR, HER2neu) negative status, basal-like phenotype, BRCA1 mutation, positive metastatic lymph nodes positive, high grading, ki67 and mitotic index [4, 10, 14–17]. Of note, Kondi-Pafiti et al. observed that EBCCs-c-KitR are decreased in IBC and they appeared to be a marker of high-grade IBC, correlating with poor prognosis [18]. In particular it has been demonstrated that c-KitR may be expressed in breast myoepithelial cells and sometimes in some macrophages and smoothmuscle cells of vascular walls. Amin et al. evaluated the relationship between EBCCs-c-KitR and microvascular density (MVD), showing that the loss of EBCCs-c-KitR is associated with high MVD and some markers of tumor aggressiveness (higher tumor grade, larger size, and more lymph node metastasis) [3]. In this context there are no studies evaluating the correlation between c-KitR-MCs and MVD in BC patients. Therefore, we aimed at verifying the presence of a correlation between MCs-c-KitR and EBCCs-c-KitR, MCDPT, MVD to each other in 121 female early BC patients. Our results demonstrated a strong and significant correlation between MVD, MCs-c-KitR and MCDPT. With reference to this finding, it must be considered that the number of c-KitR expressing cells may be slightly greater than MCDPT in that several stromal cells (such as myoepithelial cells, macrophages and smooth muscle cells) can also result positive to c-KitR expression. Moreover, we observed a significant difference in terms of MCDPT, MVD, MCs-c-KitR, EBCCs-c-KitR vs NBE-c-KitR (*p* = 0.02) in tumor tissue vs adjacent normal tissue.

**Table 2:** MCDPT, MVD, MCs-c-KitR and EBCCs-c-KitR means ± 1 standard deviations in a series of 121 breast cancer patients

MCDPT 400x magnification (0.19 mm^2^ area)	MVD 400x magnification (0.19 mm^2^ area)	MCs-c-KitR 400x magnification (0.19 mm^2^ area)	EBCCs-c-KitR 400x magnification (0.19 mm^2^ area)
^a^7.49 ± 2.81	^a^29.41 ± 6.63	^a^8.75 ± 3,26	^a^32.98 ± 16.61
^a^2,86 ± 1,24	^a^12,38 ± 3,97	^a^3,01 ± 1,57	^a^59,69 ± 27,35
*t*-test *p* = 0.001	*p* = 0.003	*p* = 0.001	*p* = 0.02

^a^Mean ± 1 standard deviation.

Taken together, the above evidences confirm the involvement of both MC tryptase and MCs-c-KitR in BC tumor angiogenesis. Additionally, the lack of correlation between increased MVD and high percentage of EBCCs-c-KitR suggests that clones of malignant EBCCs positive to c-KitR probably are not involved in tumor angiogenesis. Therefore, c-KitR and tryptase expressing MCs could represent a novel surrogate angiogenic marker in BC patients. Finally, they could also be identified as a new potential anti-angiogenetic target for several c-KitR-TK inhibitors (e.g. masitinib, sunitinib) or tryptase inhibitors (e.g. nafamostat mesilate, gabexate mesilate).

## MATERIALS AND METHODS

The clinico-pathological features of the patients are summarized in the Table 1. A series of 121 BC patients observed at the Clinical Surgery Unit of the “Magna Graecia” University of Catanzaro were selected. Biopsy specimens were collected from 121 female BC patients who had undergone BC surgery. Patients were selected accordingly the presence of a primary, invasive breast tumor (stage T1-T3), the presence or not of metastases in axillary lymph nodes (stage N0-N2), the absence of distant metastases (M0), the presence of unilateral breast cancer and the absence of previous or concomitant primary cancer. Patients were staged according to the International Union Against Cancer Tumor Node Metastasis (UICC-TNM) classification [31]. They not received neo-adjuvant therapies. Surgical treatment performed was either a modified radical mastectomy (44 patients in which the tumor had a diameter > 3 cm) or a quadrantectomy with axillary lymphadenectomy. No patient was subjected to the investigation of sentinel lymph node. Following surgery, a course of 5–6 weeks of radiation therapy (77 patients) was performed. On the basis of clinico-pathological features patients were evaluated to receive adjuvant hormonal therapy or chemotherapy or both. Full ethical approval and signed consent from individual patients were obtained to conduct the study. The full name of ethics institutional committee review board that approved our study is: University Hospital Ethics Committee “Mater Domini”, Germaneto, Catanzaro, Italy.

The histological diagnosis was made on haematoxylineosin-stained slides and histopathological grading was performed according to the criteria described by Bloom and Richardson, as well, moderately and poorly differentiated state [32]. For the evaluation of MCDPT, MVD, MCs-c-KitR and EBCCs-c-KitR a three-layer biotin-avidin-peroxidase system was utilized [33]. Briefly, six-μm-thick serial sections of formalin-fixed and paraffin-embedded of tumor tissue and adjacent normal breast tissue were cut. Then, sections were microwaved at 500 W for 10 min, after which endogenous peroxidase activity was blocked with 3% hydrogen peroxide solution. Adjacent sections were stained with human-specific monoclonal antibodies anti-tryptase (clone AA1; Dako, Glostrup, Denmark) diluted 1:100 for 1 h at room temperature, with anti-CD34 (QB-END 10; Bio-Optica Milan, Italy) as pan-endothelial marker diluted 1:50 for 1 h at room temperature and with the rabbit polyclonal antibodies anti-CD117 to c-KitR (Dako, Glostrup, Denmark) diluted 1:100 at for 1 h at room temperature [2]. The bound antibody was visualized using a biotinylated secondary antibody, avidin-biotin peroxidase complex and fast red. Nuclear counterstaining was performed with Gill's haematoxylin no. 2 (Polysciences, Warrington, PA, USA). The primary antibody was omitted in negative controls.

An image analysis system (Quantimet500 Leica, Wetzlar, Germany) was utilized [33]. For tumor tissue the five areas with higher immunostaining (‘hot spots’) were selected at low magnification and individual MCDPT (Figure 1A), MVD (Figure 1B), MCs-c-KitR (Figure 1C) and EBCCs-c-KitR (Figure 1D) were counted at x400 magnification (0.19 mm2 area).

With special reference to MVD, each microvessel was defined as single brown stained endothelial cells, endothelial cell clusters and microvessels, clearly separated from adjacent microvessels, tumor cells and other connective tissue elements were counted [54]. In the same manner, in adjacent normal tissue the five areas with higher immunostaining (‘hot spots’) were selected at low magnification and individual MCDPT (Figure 2A), MVD (Figure 2B), MCs-c-KitR (Figure 2C) and NBE-c-KitR (Figure 2D) were counted at x400 magnification (0.19 mm2 area).

MCDPT, MVD, MCs-c-KitR, EBCCs-c-KitR and NBE-c-KitR mean values ±1 standard deviation (s.d.) were evaluated by two independent observers (V.Z. and G.R.) for each tumor sample and in all series of sections (Table 2). Correlations between MCDPT, MVD, MCs-c-KitR and EBCCs-c-KitR were calculated using Pearson's (r) analysis (Figure 3A, 3B). Difference between mean regarding tumor tissue vs normal tissue in terms of MCDPT (*p* = 0.001), MVD (*p* = 0.003) MCs-c-KitR (*p* = 0.001), EBCCs-c-KitR vs NBE-c-KitR (*p* = 0.02) was evaluated by *t*-test analysis. Obtained data evaluated by *t*-test analysis show a significant difference between mean regarding tumor tissue vs normal tissue in terms of MCDPT (*p* = 0.001), MVD (*p* = 0.003) MCs-c-KitR (*p* = 0.001), EBCCs-c-KitR (*p* = 0.02) as summarized in Table 2.

The correlations between the above indexes and the clinico-pathological features listed in Table 1 were analyzed by the Chi-square test. All statistical analyses were performed with the SPSS statistical software package (SPSS, Inc., Chicago, IL).
